# Genomic preselection with genotyping-by-sequencing increases performance of commercial oil palm hybrid crosses

**DOI:** 10.1186/s12864-017-4179-3

**Published:** 2017-11-02

**Authors:** David Cros, Stéphanie Bocs, Virginie Riou, Enrique Ortega-Abboud, Sébastien Tisné, Xavier Argout, Virginie Pomiès, Leifi Nodichao, Zulkifli Lubis, Benoit Cochard, Tristan Durand-Gasselin

**Affiliations:** 1 CIRAD, UMR AGAP (Genetic Improvement and Adaptation of Mediterranean and Tropical Plants Research Unit), F-34398 Montpellier, France; 2 South Green Bioinformatics Platform, Montpellier, France; 3 P.T. SOCFINDO Medan, Medan, 20001 Indonesia; 4 INRAB, CRAPP, Pobè, Benin; 5 PalmElit SAS, 34980 Montferrier sur Lez, France

**Keywords:** Genetic gain, Genomic selection, Genotyping-by-sequencing, Hybrid, Oil palm, Reciprocal recurrent selection

## Abstract

**Background:**

There is great potential for the genetic improvement of oil palm yield. Traditional progeny tests allow accurate selection but limit the number of individuals evaluated. Genomic selection (GS) could overcome this constraint. We estimated the accuracy of GS prediction of seven oil yield components using A × B hybrid progeny tests with almost 500 crosses for training and 200 crosses for independent validation. Genotyping-by-sequencing (GBS) yielded +5000 single nucleotide polymorphisms (SNPs) on the parents of the crosses. The genomic best linear unbiased prediction method gave genomic predictions using the SNPs of the training and validation sets and the phenotypes of the training crosses. The practical impact was illustrated by quantifying the additional bunch production of the crosses selected in the validation experiment if genomic preselection had been applied in the parental populations before progeny tests.

**Results:**

We found that prediction accuracies for cross values plateaued at 500 to 2000 SNPs, with high (0.73) or low (0.28) values depending on traits. Similar results were obtained when parental breeding values were predicted. GS was able to capture genetic differences within parental families, requiring at least 2000 SNPs with less than 5% missing data, imputed using pedigrees. Genomic preselection could have increased the selected hybrids bunch production by more than 10%.

**Conclusions:**

Finally, preselection for yield components using GBS is the first possible application of GS in oil palm. This will increase selection intensity, thus improving the performance of commercial hybrids. Further research is required to increase the benefits from GS, which should revolutionize oil palm breeding.

**Electronic supplementary material:**

The online version of this article (10.1186/s12864-017-4179-3) contains supplementary material, which is available to authorized users.

## Background

Genomic selection (GS) is an efficient method of marker-assisted selection to improve quantitative traits [1]. It uses a statistical approach that gives the genomic estimated genetic value of the candidates for selection usually without phenotypic data records but genotyped at high marker density. The prediction model is calibrated with the phenotypic data records and the genotypes of individuals that formed the “training set”. The key factor that determines the way GS can be implemented in practice is its accuracy, which is defined as the correlation between the predicted and the true (unknown) genetic value of the candidates for selection. The commercial cultivars of many plant species are hybrids [2]. Empirical estimates of GS accuracy for hybrids have been obtained, particularly for major crops including maize, rice, and wheat [3–7], showing the good potential of GS for hybrid breeding (see Zhao et al. [8] for a review).

Cultivated oil palm (*Elaeis guineensis*) is a hybrid crop and is among the most economically important agricultural plant species in the world. Palm oil production is currently over 60 Mt. [9], making it the number one vegetable oil worldwide. There is high potential for the genetic improvement of yield in oil palm, as it has only been the subject of a few generations of modern breeding. Breeding of this diploid species relies on sexual reproduction and reciprocal recurrent selection (RRS) between heterotic groups A (mostly from Asia) and B (from Africa). The commercial cultivars are A × B hybrids [10, 11]. Candidates for selection are traditionally evaluated in progeny tests to estimate their general combining abilities (GCAs, i.e., half breeding values in hybrid crosses), upon which the selection is based. Progeny tests are required as some yield components have a low heritability [12], and because parental performances may be poor indicators of the performance of the hybrid due to gene-frequency differences between parental populations and non-additive effects [13, 14]. The progeny tests enable highly accurate selection, but also have drawbacks that constrain the rate of genetic gain. Indeed, they lead to a selection phase that takes place years after reproductive maturity, thereby increasing the generation interval to around 20 years. In addition, the difficulty and costs associated with these long term evaluations limit the number of individuals evaluated, resulting in low selection intensity. In this context, the potential of GS for palm oil yield is high, and has been confirmed in the few previous studies dealing with GS in this species [15–19].

Nevertheless, the empirical GS studies published in oil palm so far [17–19] suffered from some limitations. First, like in many studies in other crops, they used single datasets which can bias accuracies upwards [20–22]. Also, using the common cross-validation approach to estimate GS accuracy with a single dataset, like in Cros et al. [17], artificially reduces the size of the training set compared to when the method is implemented in practice. Indeed, this approach requires that individuals be left out of the training set so they can be used for validation, whereas in real life, the genomic model would be trained using the whole dataset. In addition, Cros et al. [17] and Marchal et al. [18] used low density genome coverage with simple sequence repeat markers (SSR), whereas a high throughput genotyping method would be required for large scale genotyping of selection candidates. Genotyping-by-sequencing (GBS) is an appropriate and cost effective way to achieve this goal [23]. GBS relies on the sequencing of the genome regions delimited by the restriction site of enzymes used to reduce genome complexity [24]. GBS enables multiplexed sequencing and can easily be applied to large populations, scoring thousands of SNPs. In oil palm, the only study using GBS indicated it was an efficient genotyping approach for quantitative trait loci (QTL) detection [25]. Finally, as in many GS papers, these empirical studies only focused on GS accuracy and did not investigate the additional gain that could result from the actual use of GS in a breeding scheme.

The present study had two goals: (i) to obtain empirical GS prediction accuracies for unobserved oil palm hybrid crosses that are more relevant for the practical implementation of the method than the previous accuracies published for this species, and (ii) to quantify the additional genetic gain that could have been obtained in the hybrid crosses selected from the validation progeny tests if the A and B parental populations had previously undergone genomic preselection.

In detail, to reach our first goal, we trained the GS model with the data of all the individuals progeny tested in the most recent breeding cycle of the PalmElit commercial breeding program, and validated it with an independent dataset of progeny tests. The phenotypic data were collected in field trials comprising A × B oil palm hybrid crosses planted in two sites in Indonesia. The almost 500 hybrid crosses planted in Site 1 were used to train the GS model that predicted the genetic value of around 200 crosses planted in Site 2, as well as the GCAs of their 67 A and 42 B parents (validation sets). The study considered seven traits that are key components of palm oil yield. The A and B parents of the crosses were genotyped by GBS, resulting in 5092 SNPs in Group A individuals and 8311 SNPs in Group B. To reach our second goal, we considered FFB (fresh fruit bunches, or annual cumulative bunch production) and used the empirical values estimated in Site 2 for this trait, i.e. the prediction accuracies of genomic and phenotypic selection and the genetic variances of parental populations. From these values, we ran a simulation in which 5000 A and 5000 B individuals were subjected to two breeding approaches: a conventional RRS mimicking the actual implementation of Site 2 progeny tests, i.e. 125 individuals chosen per group (without preselection on FFB) and progeny tested, and an alternative genomic approach in which the 125 progeny tested individuals per group were preselected on their genomic estimated GCA for FFB.

## Methods

### General overview

The empirical estimation of the GS prediction accuracies took place in two steps: (i) computation of reference cross values and reference parental GCAs from Site 2 using TBLUP, traditional (i.e. pedigree-based) best linear unbiased predictor methodology, and (ii) prediction of these reference values with the genomic BLUP (GBLUP) GS mixed model, and with PBLUP, a pedigree-based model used as control.

In the first step, reference cross values were obtained from the observed cross values with adjustment to remove the effects of the experimental design (trial, block, etc.). For this purpose, we used the phenotypic data from Site 2 and a linear mixed model (TBLUP, see mixed model analyses section below). The reference cross values were defined as the sum of the GCA of their A and B parents and the specific combining ability (SCA, corresponding here to the dominance effect) of the crosses. Here, the goal was to obtain reference cross values as close as possible to the observed values avoiding any possible bias due to the experimental design. For this reason, relationships between parents were not taken into account (i.e. we assumed independence among the parents of each group and among hybrid crosses) to avoid shrinkage of the parental GCAs towards family means. The reference parental GCAs were obtained with the same model but using genealogical coancestries (i.e. kinships) between parents, i.e. in the same way as the GCAs were obtained before the genomic era [26]. The reference cross values and the reference parental GCAs were the values to be predicted with GBLUP and PBLUP in the second step.

In the second step, GBLUP was used with the phenotypic data from Site 1 and molecular data from Sites 1 and 2 to predict the cross value of the hybrid crosses at Site 2, as well as the GCAs of their A and B parents. PBLUP was applied like GBLUP but using pedigree data instead of molecular data when computing coancestries between individuals. The goal of PBLUP was to assess the usefulness of marker data, in particular their ability to capture genetic differences within full-sib families of parents (i.e. Mendelian segregation terms), which is necessary because the aim of oil palm breeding is to select the best individuals in the best families [27]. As PBLUP only used pedigrees to model genetic covariances between training and validation individuals, it cannot account for the Mendelian sampling term and predicted identical GCAs to parental full-sibs in the test set. Thus, PBLUP only differentiated parental families, not individuals within families. PBLUP was therefore used as a control method to allow us to check whether GBLUP is able to account for Mendelian sampling terms in addition to family effects. The A and B parents of the training and validation crosses formed two complex populations with high relatedness but with some variation in this parameter, which reflects the way GS could be implemented in oil palm to predict the cross value of hybrids obtained by mating individuals that could be related to the training individuals to different degrees (full-sibs, half-sibs, progeny, cousins, etc.).

Two types of GS prediction accuracies were computed: (i) the prediction accuracy of cross values, defined as the correlation between the reference value of the hybrid crosses and their genomic estimated value, and (ii) the prediction accuracy of GCAs in each parental group, equal to the correlation between the reference GCAs and the genomic estimated GCAs.

From these results, we simulated large A and B populations of selection candidates (with genetic variances obtained from Site 2 data) and two breeding approaches aiming at improving FFB in hybrid crosses. First, we simulated the conventional RRS methodology that was used to set up Site 2 progeny tests, in which progeny tested A and B individuals can be considered as random samples of the populations of candidates in terms of FFB (i.e. no preselection for this trait), and GCA selection accuracies are high (with actual values obtained from TBLUP analyses at Site 2). Second, we simulated RRS with genomic preselection prior to progeny tests, using the GS prediction accuracies obtained in the first part of the study.

### Breeding populations

Group A was mostly made up of Deli individuals. The Deli breeding population originated from four ancestral oil palms planted in 1848 in Indonesia. The population was selected for yield at least in the early twentieth century and inbreeding was commonly used, by selfing or by mating related selected individuals [12]. Group A also included individuals from the Angola population resulting from material collected before the 1950s. Group B was made up of several breeding populations of African origin. The La Mé population originated from Côte d’Ivoire, Yangambi and Lisombe Kinshasa from Democratic Republic of the Congo, Sibiti from Republic of the Congo (although related to Yangambi). The African populations also derived from a few founders collected during the first half of the twentieth century. In particular, the La Mé population originated from three individuals and the Yangambi and Sibiti populations originated from about 10 individuals [28]. African populations were also subject to inbreeding and selection for yield. Additional file 1: Table S1 lists the number of A and B individual progeny tested per parental group in the two sites, as well as their status (genotyped or not, present in only one site or in both). Regarding the training crosses, the 150 Group A parents were from Deli (139) and Angola (11) populations, and the 156 Group B parents originated from La Mé (112), Yangambi (24), Lisombe Kinshasa (8), La Mé × Yangambi / Sibiti (7) and Nigeria × Yangambi (5). Regarding the validation crosses, the 67 Group A parents were of Deli (60) and Angola × Deli (7) origin, and the 42 Group B parents of La Mé (18), Yangambi (15), Nigeria × Yangambi (4), La Mé × Yangambi / Sibiti (4) and Lisombe Kinshasa (1). The pedigrees were known over several generations, and are shown in Additional files 2 and 3: Figures S1 and S2, for groups A and B, respectively (with training individuals in blue and validation individuals in red). In Group A, the mean maximum genealogical coancestry [29] between each validation individual and the training individual (*f*_*max* V-T_), ranged from 0 to 0.75 (average 0.25), and 60% of the validation individuals had a *f*_*max* V-T_ of 0.25, i.e. had at least one full-sib or a first generation parent in the training set. In Group B, *f*_*max* V-T_ ranged from 0 to 0.77 (average 0.49). The validation sets were therefore closely related to the training set, which corresponds to the way GS would be applied in oil palm breeding to predict the breeding value of individuals of the same generation or of the following generation compared to the progeny tested individuals (or the genetic values of crosses between them).

Table 1 shows the distribution of crosses among Group A × Group B populations.

**Table 1:** Distribution of crosses among Group A × Group B populations in both sites

		Site 1 (training)		Site 2 (validation)	
		Group A		Group A	
		Angola	Deli	Angola × Deli	Deli
Group B	Lisombe Kinshasa	0	28	0	2
	La Mé	34 (33)	294 (285)	17	88
	La Mé × Yangambi / Sibiti	0	25	0	8
	Nigeria	0	18	0	0
	Nigeria × La Mé	0	0	0	9
	Yangambi	0	98	10	65 (64)
TOTAL		497 (487)		199 (198)	

### Experimental design

Both sites are located in North Sumatra, on the SOCFINDO Aek Loba estate. Site 1 (Aek Loba Timur) is located 2° 39′ North – 99° 42′ East and Site 2 (Aek Kwasan division VI) 2° 38′ North – 99° 37′ East, at a distance of around 9 km between the two sites. Both sites are located around 50 m above sea level. They both have deep well drained soils developed over reworked Toba Tuffs (haplic arenosols and dystric cambisol types in Site 1 and haplic acrisols type in Site 2). The same standard cultural practices were used in both sites, and the same protocol was used for recording data.

The crosses were A × B hybrids planted in fields from 1986 to 2003 (Site 1) and from 2005 to 2010 (Site 2), in standard trials for the evaluation of oil palm crosses, i.e. randomized complete block designs with five or six blocks, or in balanced lattices of rank four or five. Additional file 1: Table S1 summarizes the characteristics of the experimental designs in the two sites. The data from Site 1 are also described in detail in Cros et al. [17] and Marchal et al. [18]. The total number of crosses in Site 2 was 433 with data for bunch production (393 for bunch quality) but only 199 (198) were used as the validation set, the others being excluded because they were also present in Site 1 (9 crosses) or because their parents were not genotyped. All the data used were collected on tenera (thin-shelled commercial type) individuals. The crosses were produced by mating A and B parents planted at SOCFINDO or at CRAPP (Pobè, Bénin) using incomplete factorial mating designs. The plant material belongs to the PalmElit (www.palmelit.com) breeding program. PalmElit is a leading oil palm breeding and seed production company.

### Phenotypic data

Phenotypic data on hybrid individuals were available for three bunch production traits: annual cumulative bunch production (FFB, in kg), annual cumulative bunch number (BN) and annual average bunch weight (ABW, in kg) and for four bunch quality traits: fruit-to-bunch ratio (FB, in %), pulp-to-fruit ratio (PF, in %), oil-to-pulp ratio (OP, in %) and oil extraction rate (OER, in %). OER is the percentage of oil in the bunch and is the product of FB, PF and OP. FFB and BN data were collected at 10 day intervals. ABW is obtained by dividing FFB by BN. Data from palms aged three to seven were used for bunch production traits. Data from palms aged five and six were used for bunch quality traits. The coefficients of variation and skewness (asymmetry of the distribution) of cross values adjusted for experimental design were similar among sites for all traits (Table 2), indicating that phenotypic variation was consistent across sites. Between-site phenotypic correlations were estimated on the nine crosses common to both sites. The correlations were on average 0.78, ranging from 0.45 (FB) to 0.96 (BN), indicating that genotype-by-environment interactions were negligible.

**Table 2:** Coefficient of variation (CV) and skewness in the two sites and between-site correlations for the reference (adjusted for experimental design) cross values of the seven traits studied. Correlations were computed over the nine crosses common to both sites

	ABW	BN	FFB	FB	PF	OP	OER
CV Site 1	9.0%	8.3%	5.8%	2.9%	3.8%	3.5%	6.4%
CV Site 2	9.0%	9.3%	5.0%	1.1%	1.7%	1.7%	2.7%
Skewness Site 1	1.44	−0.67	0.01	−0.34	−0.24	−0.26	−0.44
Skewness Site 2	1.47	−0.24	−0.22	−0.07	−1.00	−0.34	−0.65
Correlation	0.85	0.96	0.81	0.45	0.76	0.94	0.63

### Molecular data

DNA extraction was performed by ADNid (www.adnid.fr) on lyophilized tissue from the youngest opened leaf of each individual, using a modified mixed alkyltrimethylammonium bromide (MATAB) protocol. GBS was conducted on the DNA extracts by a company called DArT (www.diversityarrays.com) using their DArTseq™ protocol [30], which combined complexity reduction of the genome and next generation sequencing [24, 31]. DNA samples were processed in digestion/ligation reactions mainly as per Kilian et al. [30] but using two adaptors corresponding to the *Pst*I and *Hha*I restriction enzyme overhangs and moving the assay on the sequencing platform as described by Sansaloni et al. [32]. The *Pst*I-compatible adapter was designed to include the Illumina flowcell attachment sequence, the sequencing primer sequence and the “staggered”, varying length barcode region, similar to the sequence reported by Elshire et al. [24]. The reverse adapter contained the flowcell attachment region and the *Hha*I-compatible overhang sequence. Only *Pst*I-*Hha*I mixed fragments were effectively amplified in 30 rounds of PCR using the following reaction conditions: (1) 94 °C for 1 min, (2) 30 cycles at 94 °C for 20 s, 58 °C for 30 s, 72 °C for 45 s and (3) 72 °C for 7 min. Next, PCR equimolar amounts of amplification products from each sample in the 96-well microtiter plate were bulked and applied to c-Bot (Illumina) bridge PCR followed by sequencing on Illumina HiSeq2500. Single read sequencing was run for 77 cycles.

The GBS analysis pipeline implemented in Tassel GBS version 5.2.29 [33] was used to call SNPs according to the parameters listed in Additional file 4: Table S2. From the total number of good barcoded reads (152,020,019 out of 238,493,056), the pipeline found 476,589 tags, aligned with Bowtie2 software. The tag mapping and the polymorphism calling identified 109,201 polymorphic sites. The data were further processed with VCFtools [34]. Indels and SNPs that were not biallelic were discarded. Data points with a sequencing depth of less than five were set to missing. SNPs with more than 50% missing data were discarded. Using a custom R script [35], the SNPs appearing as outliers in terms of mean depth (i.e. higher than 500) were discarded, as it was assumed this could indicate duplication in the genome. This resulted in 19,432 SNPs. The molecular dataset was split into two, one for Group A and the other for Group B. The SNPs that mapped on the unassembled part of the genome were discarded, as the imputation of sporadic missing data required known positions. Mendelian segregation between parents and offspring was checked and the inconsistent data points were set to missing. The SNP homozygotes or with more than 5% of Mendelian inconsistencies in a parental group were discarded from this group. This resulted in 5092 SNPs in Group A and 8311 in Group B.

**Fig. 1:**
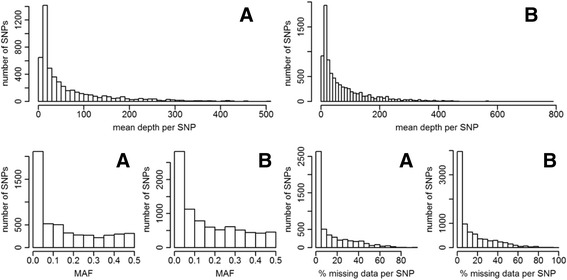
Distribution of mean depth per SNP (top), SNPs minor allele frequency (MAF) (bottom left), and percentage of missing data per SNP (bottom right) in Group A and Group B

The average distance between adjacent SNPs was 132,297 bp in Group A and 81,289 in Group B. Additional files 5 and 6: Figures S3 and S4 show the physical map of the SNPs in the two groups. The depth per data point was on average 69.8 in Group A (range 5–2588) and 78.9 in Group B (5–3177). The mean depth per SNP was 65.4 (7–507) in Group A and 74.5 (6.9–782.6) in Group B. The mean minor allele frequency (MAF) was 0.15 (0.002–0.5) in Group A and 0.16 (0.003–0.5) in Group B. The percentage of missing data was on average 13.2% (0%–90.4%) in Group A and 14% (0%–96.3%) in Group B (see distributions in Fig. 1). Missing SNP data was imputed with BEAGLE 4.0 [36, 37], with the following parameters: burn in-its = 6, impute-its = 15, window = 300 and overlap = 10.

Molecular coancestries between genotyped individuals were calculated according to Lynch [38] and Li et al. [39]. For each validation individual, the maximum coancestry with the training individuals was computed, and the mean value over all the validation individuals was calculated.

### Mixed model analyses

The different mixed models (TBLUP, GBLUP and PBLUP) were of the form:

1 $$ \mathbf{Y}=\mathbf{X}\boldsymbol{\upbeta } +\mathbf{Zb}+{\mathbf{Z}}_{\mathbf{A}}{\mathbf{g}}_{\mathbf{A}}+{\mathbf{Z}}_{\mathbf{B}}{\mathbf{g}}_{\mathbf{B}}+{\mathbf{Z}}_{\mathbf{D}}{\mathbf{s}}_{\mathbf{A}\mathbf{B}}+\mathbf{e} $$

where **Y** is the vector of the phenotypes of the hybrid individuals, **β** and **b** are the vectors of fixed and random effects due to the experimental design, respectively, **X** and **Z** their associated incidence matrices, **g**_**A**_ and **g**_**B**_ are the vectors of GCA of parents A and B, respectively, **s**_**AB**_ is the vector of SCA of crosses, **Z**_**A**_, **Z**_**B**_ and **Z**_**D**_ their incidence matrices and **e** is the vector of residual effects. The random genetic effects followed the model of Stuber and Cockerham [40] for hybrid crosses, with **g**_**A**_ ~ N(0, ${\sigma}_{g_A}^2$ × **Γ**_**A**_), **g**_**B**_ ~ N(0, ${\sigma}_{g_B}^2$ × **Γ**_**B**_) and **s** ~ N(0, ${\sigma}_d^2$ × **Γ**_**D**_ **)**, where ${\sigma}_{g_A}^2$ and ${\sigma}_{g_B}^2$ are the additive variances of the A and B parents in A × B hybrid crosses, respectively, ${\sigma}_d^2$ is the variance of the dominance effects in the A × B population, and **Γ**_**A**_, **Γ**_**B**_ and **Γ**_**D**_ are the matrices of known constants used to define the covariance among GCAs of a given parental group and between SCAs (see below).

Fixed effects were overall mean, “trial”, “block” and, for bunch production traits, “age”. Random effects associated with the experimental design were “elementary plots”, “individual” and, for bunch production traits, interaction “age*cross” (“α*s_**AB**_”) and, for lattice trials, “incomplete block”. The “incomplete block” and “elementary plot” effects were nested in “block” and “trial”. The random experimental design effects followed a normal distribution N(0, *σ* ^2^ × **I**), where **I** is the identity matrix and *σ* ^2^ the associated variance, with the exception of α*s_**AB**_ that followed N(0, ${\sigma}_{\alpha \ast d}^2$ × **I** ⊗ **Γ**_**D**_). The errors **e** followed N(0, ${\sigma}_e^2$ × **I**), where ${\sigma}_e^2$ is the residual variance.

Variance parameters were estimated by restricted maximum likelihood (REML) and solutions of the mixed models were obtained by resolving Henderson’s mixed model eqs. [41], using R-ASReml version 3.0 [35, 42].

### Computation of reference cross values and reference GCAs for site 2 (TBLUP)

When TBLUP was used to estimate reference hybrid cross values, **Γ**_**A**_ and **Γ**_**B**_ were identity matrices (**Γ**_**A**_ **= I**, **Γ**_**B**_ **= I**).

When TBLUP was used to estimate reference parental GCAs, **Γ**_**A**_ **=** 0.5**A**_**A**_ and **Γ**_**B**_ **=** 0.5**A**_**B**_, where **A**_**A**_ and **A**_**B**_ were the genealogical relationship matrices computed from the pedigree of the group, with elements 2*f*_*xy*_, where *f*_*xy*_ is the coefficient of coancestry between individuals *x* and y. With TBLUP, the **Y** vector contained the phenotypic data of Site 2 and the **Γ**_**A**_ and **Γ**_**B**_ matrices included only the individuals progeny tested in Site 2.

The dominance relationship matrix **Γ**_**D**_ was obtained as **Γ**_**D**_ = **Γ**_**A**_ ⊗ **Γ**_**B**_, i.e. with elements **Γ**_**D***ab,a’b’*_ *=* {**Γ**_**A***aa’*_ **Γ**_**B***bb’*_} giving the coefficient of fraternity between two hybrid crosses involving *a* and *a’* parents from Group A and *b* and *b’* parents from Group B (i.e. crosses *a* × *b* and *a’* × *b’*), as A and B individuals are not related [40, 43].

The reference cross value for cross *a* × *b* was obtained from the solutions of the mixed model, as ${\widehat{\mathbf{g}}}_{\mathbf{A}\left(\mathbf{a}\right)}+{\widehat{\mathbf{g}}}_{\mathbf{B}\left(\mathbf{b}\right)}+{\widehat{\mathbf{s}}}_{\mathbf{A}\mathbf{B}\left(\mathbf{a}\mathbf{b}\right)}$.

The proportion of dominance variance between crosses over the total genetic variance between crosses was calculated for Site 2 from the TBLUP model including genealogical information. It was obtained as the ratio of SCA variance to the sum of SCA and GCA variances: $\left( Tr\left({\boldsymbol{\Gamma}}_{\boldsymbol{D}}\right)/{n}_d\right){\sigma}_d^2/\left(\left( Tr\left({\boldsymbol{\Gamma}}_{\boldsymbol{D}}\right)/{n}_d\right){\sigma}_d^2+\left( Tr\left({\boldsymbol{\Gamma}}_{\boldsymbol{A}}\right)/{n}_A\right){\sigma}_{g\mathrm{A}}^2+\left( Tr\left({\boldsymbol{\Gamma}}_{\boldsymbol{B}}\right)/{n}_B\right){\sigma}_{g\mathrm{B}}^2\right)$, with *n*_*A*_, *n*_*B*_ and *n*_*D*_ the order of the matrices **Γ**_**A**_, **Γ**_**B**_ and **Γ**_**D**_, respectively.

### Genomic prediction model (GBLUP) and control pedigree-based model (PBLUP)

By contrast with TBLUP, the **Y** vector used in GBLUP and PBLUP contained the phenotypic data from Site 1, and the **Γ**_**A**_ and **Γ**_**B**_ matrices included the individuals progeny tested in both Site 1 and Site 2.

For PBLUP, we used **Γ**_**A**_ **=** 0.5**A**_**A**_ and **Γ**_**B**_ **=** 0.5**A**_**B**_.

For GBLUP, the **Γ**_**A**_ and **Γ**_**B**_ matrices were obtained from marker data but as some progeny tested individuals were not genotyped, their pedigree coancestry had to be combined with the molecular coancestry of the genotyped individuals. For parental Groups A and B, **Γ.** inverse (with . denoting the parental group) was built as follows [44, 45]:

$$ \boldsymbol{\Gamma} {.}^{-1}={\left(0.5\mathbf{A}.\right)}^{-1}+\left[\begin{array}{cc}0& 0\\ {}0& \mathbf{G}{.}^{-1}-{\left(0.5\mathbf{A}{.}_{22}\right)}^{-1}\end{array}\right] $$

where **G.** is the genomic coancestry matrix and **A.**_**22**_ the genealogical relationship matrix of the genotyped individuals. The additive genomic relationship matrices **G.** were computed according to the standard approach of VanRaden [46] and Habier et al. [47].

The dominance relationship matrices **Γ**_**D**_ were obtained as previously described (**Γ**_**D**_ = **Γ**_**A**_ ⊗ **Γ**_**B**_).

In order to investigate the usefulness of estimating the dominance effects (SCAs), the predicted cross values where obtained with or without SCA, i.e. for a cross *a* × *b*, the predicted cross value was (i) ${\widehat{\mathbf{g}}}_{\mathbf{A}\left(\mathbf{a}\right)}+{\widehat{\mathbf{g}}}_{\mathbf{B}\left(\mathbf{b}\right)}+{\widehat{\mathbf{s}}}_{\mathbf{A}\mathbf{B}\left(\mathbf{a}\mathbf{b}\right)}$ or (ii) ${\widehat{\mathbf{g}}}_{\mathbf{A}\left(\mathbf{a}\right)}+{\widehat{\mathbf{g}}}_{\mathbf{B}\left(\mathbf{b}\right)}$.

### Prediction accuracies

The validation crosses were initially divided into six random sets of equal size, and for each validation set, the prediction accuracy for cross values was obtained as the Pearson correlation between the reference and the predicted cross values. GCA prediction accuracy was obtained as the Pearson correlation between the reference and the predicted GCAs on the 67 Group A individuals and 42 B available for validation, with no replicates due to small population sizes.

### Varying factors affecting prediction accuracies

In order to investigate the effect of taking pedigree information into account when imputing the missing molecular data with BEAGLE 4.0, the imputation step was carried out with and without the pedigrees. In order to assess whether using the pedigree affected prediction accuracy, a Student’s t-test with paired data was performed for each trait on the six replicates of the cross value prediction accuracies obtained with all SNPs.

To investigate the effect of marker density on prediction accuracy, we varied the number of SNPs used to construct the genomic matrices of GBLUP from 200 SNPs to 3000 (using the same number simultaneously in both parental groups). For a given level of SNP density, we made 26 replicates of random samples of SNPs, using the same replicates for all the traits. To assess if GBLUP and PBLUP led to significant differences in cross values prediction accuracies, an analysis of variance (ANOVA) was performed for each trait and SNP density with the following factors: prediction method (GBLUP and PBLUP), set of hybrid crosses (from one to six) and, for GBLUP, replicates of the SNP subset (from 1 to 26).

In order to study whether filtering SNPs based on their percentage of missing data affected prediction accuracies, the variation in the number of SNPs was also implemented using the SNPs with the lowest percentage of missing data, with three replicates of random SNPs with same percentage of missing data for each level of numbers of SNP. An ANOVA similar to the one explained above was performed to assess whether the effect of SNP filtering minimized the percentage of missing data on the prediction accuracies of the cross values.

An overview of the method used to obtain the empirical GS prediction accuracies is presented in Additional file 7: Figure S5.

### Quantifying the impact of genomic preselection on hybrid performances for FFB

For each parental group, the true (**g**′), reference (${\widehat{\mathbf{g}\prime}}_{\mathbf{TBLUP}}$) and genomic estimated GCAs (${\widehat{\mathbf{g}\prime}}_{\mathbf{GBLUP}}$) of the 5000 individuals that comprised the populations of candidates were jointly simulated from a multivariate normal distribution with the *mvrnorm* R function [48], with ′ denoting the fact that these parameters relate to the populations of parents of the hybrid crosses planted in Site 2, which was used to evaluate the impact of genomic preselection. This simulation required the variance-covariance matrix between **g**′, ${\widehat{\mathbf{g}\prime}}_{\mathbf{TBLUP}}$ and ${\widehat{\mathbf{g}\prime}}_{\mathbf{GBLUP}}$ for the two groups and the mean FFB value of the hybrid crosses planted in Site 2 over the 3–7 year production period (μ_FFB_). The variance-covariance matrices were:

$$ \left(\begin{array}{ccc}{\sigma}_{g\prime}^2& Cov\left({\widehat{{\boldsymbol{g}}^{\prime}}}_{\boldsymbol{TBLUP}},{\boldsymbol{g}}^{\prime}\right)& Cov\left({\widehat{{\boldsymbol{g}}^{\prime}}}_{\boldsymbol{GBLUP}},{\boldsymbol{g}}^{\prime}\right)\\ {} Cov\left({\boldsymbol{g}}^{\prime },{\widehat{\boldsymbol{g}\prime}}_{\boldsymbol{TBLUP}}\right)& {\sigma}_{{\widehat{g\prime}}_{TBLUP}}^2& Cov\left({\widehat{\boldsymbol{g}\prime}}_{\boldsymbol{GBLUP}},{\widehat{\boldsymbol{g}\prime}}_{\boldsymbol{TBLUP}}\right)\\ {} Cov\left({\boldsymbol{g}}^{\prime },{\widehat{\boldsymbol{g}\prime}}_{\boldsymbol{GBLUP}}\right)& Cov\left({\widehat{\boldsymbol{g}\prime}}_{\boldsymbol{TBLUP}},{\widehat{\boldsymbol{g}\prime}}_{\boldsymbol{GBLUP}}\right)& {\sigma}_{{\widehat{g\prime}}_{GBLUP}}^2\end{array}\right) $$

which were obtained as described below.

The variance of the reference GCAs of the individuals tested in Site 2, ${\sigma}_{{{\widehat{\mathrm{g}}\prime}_{\mathrm{TBLUP}}}_{\mathrm{A}}}^2$ and ${\sigma}_{{{\widehat{\mathrm{g}}\prime}_{\mathrm{TBLUP}}}_{\mathrm{B}}}^2$, were the variance of the 67 and 42 GCAs estimated by the previously described TBLUP analysis of Site 2 for groups A and B parents used for validation, respectively. The variances of the genomic estimated GCAs, ${\sigma}_{{{\widehat{\mathrm{g}}\prime}_{\mathrm{GBLUP}}}_{\mathrm{A}}}^2$ and ${\sigma}_{{{\widehat{\mathrm{g}}\prime}_{\mathrm{GBLUP}}}_{\mathrm{B}}}^2$, were obtained similarly from GBLUP with all SNPs. From the TBLUP analysis, for groups A and B we computed the selection accuracy *r*(${\widehat{\mathbf{g}\prime}}_{\mathbf{TBLUP}}$, **g**′) associated with the progeny tests, i.e. the correlation between the true GCAs and their estimated values from progeny tests (i.e. reference GCAs). This was obtained as the mean selection accuracy of the 67 A and 42 B individuals, computed from the prediction error variances as described in Marchal et al. [18]. The variance of the true GCAs of the individuals tested in Site 2, ${\sigma}_{\mathrm{g}{\prime}_{\mathrm{A}}}^2$ and ${\sigma}_{\mathrm{g}{\prime}_{\mathrm{B}}}^2$, were obtained using the formula [[49], appendix 1]: ${\sigma}_{\mathrm{g}\prime}^2={\sigma}_{{\widehat{\mathrm{g}\prime}}_{\mathrm{TBLUP}}}^2/r{\left({\widehat{{\mathbf{g}}^{\prime}}}_{\mathbf{TBLUP}},{\mathbf{g}}^{\prime}\right)}^2$. The A and B GS prediction accuracies *r*(${\widehat{\mathbf{g}\prime}}_{\mathbf{GBLUP}}$, ${\widehat{\mathbf{g}\prime}}_{\mathbf{TBLUP}}$) were taken from the GBLUP results with all SNPs, and were converted into selection accuracies using the formula [[50], p. 618, [51], p. 94]: *r*(${\widehat{\mathbf{g}\prime}}_{\mathbf{GBLUP}}$, **g**′) = *r*(${\widehat{\mathbf{g}\prime}}_{\mathbf{GBLUP}}$, ${\widehat{\mathbf{g}\prime}}_{\mathbf{TBLUP}}$) / *r*(${\widehat{\mathbf{g}\prime}}_{\mathbf{TBLUP}}$, **g**′).

For RRS, the 125 A individuals and 125 B included in the progeny tests were randomly chosen from the populations of candidates. For RRS with genomic preselection, the 125 A individuals and 125 B individuals included in the progeny tests were those with the highest genomic estimated GCA among each population of candidates. To investigate how the number of A and B candidates subjected to genomic preselection affected the performance of the selected hybrids, we first considered 125 candidates per parental population and then increased the number from 250 to 5000, with a step of 250. For each level of number of candidates, 20,000 replicates were made by generating random populations of candidates for each replicate.

For both breeding schemes, we selected the 10 best A parents and 10 best B parents on their GCA for FFB estimated from the progeny tests. We considered the selected hybrids were the 100 possible A × B crosses between the selected A and B individuals, and their genetic value was computed as the sum of the true GCA of their A and B parents (after centering around zero) plus μ_FFB_. The two approaches were compared based on the performance of the hybrids selected using genomic preselection μ_SEL(RRS-GP)_ minus the performance of the hybrids selected without genomic preselection μ_SEL(RRS)_, expressed in percentage of μ_SEL(RRS)_, with the formula: 100 × (μ_SEL(RRS-GP)_ - μ_SEL(RRS)_) / μ_SEL(RRS)_.

All analyses were conducted using R software version 3.3.1 [35].

## Results

### Empirical GS prediction accuracies of unobserved oil palm hybrid crosses

Molecular coancestries between training and validation individuals were similar in Group A and Group B, the mean value of the maximum molecular coancestries between validation individuals and training individuals being 0.42 (range 0.38–0.48) in Group A and 0.44 (0.40–0.47) in Group B.

**Fig. 2:**
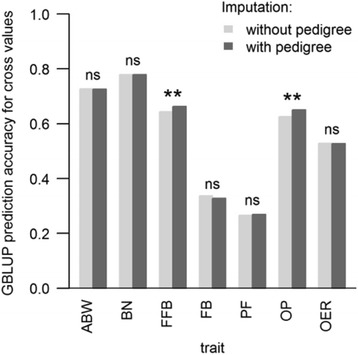
Prediction accuracies of cross values of the genomic model (GBLUP) according to the imputation method and trait concerned. Imputation was made with BEAGLE 4.0 without (light grey) and with- (dark grey) pedigrees. All SNPs were used for predictions. Significance of paired Student’s *t* test: ** *P* < 0.01, *ns* not significant. Values are means over six sets of training crosses

Using the pedigrees when imputing the missing SNP data increased the prediction accuracy of cross values for two traits, OP (oil-to-pulp ratio) and FFB (fresh fruit bunches), but did not affect the other traits (see Fig. 2, showing the results with all SNPs). For OP, the prediction accuracy increased by 4.0% (*P* = 0.007) and for FFB, by 3.1% (*P* = 0.006). For the remainder of the study, we consequently only used the molecular dataset imputed with the pedigree. In this case, GBLUP prediction accuracy for cross values using all SNPs varied from high to low depending on the trait, from 0.78 ± 0.08 (SD over the six sets of hybrid crosses) for BN (bunch number) to 0.27 ± 0.1 for PF (pulp-to-fruit ratio).

The prediction accuracies of the cross values were the same whether or not the predicted cross values included the SCA (specific combining ability) term. This was the case for all traits, marker densities and models (GBLUP and PBLUP) (not shown). The proportion of SCA variance between crosses in total genetic variance between crosses reached a maximum for FB (32.4%), followed by OER (26.0%), PF (8.9%), OP (8.5%) and ABW, BN and FFB (< 1% for the three of them). For the remainder of the study, we only present the results obtained for cross values prediction accuracies when the SCA term was not used in the prediction.

**Fig. 3:**
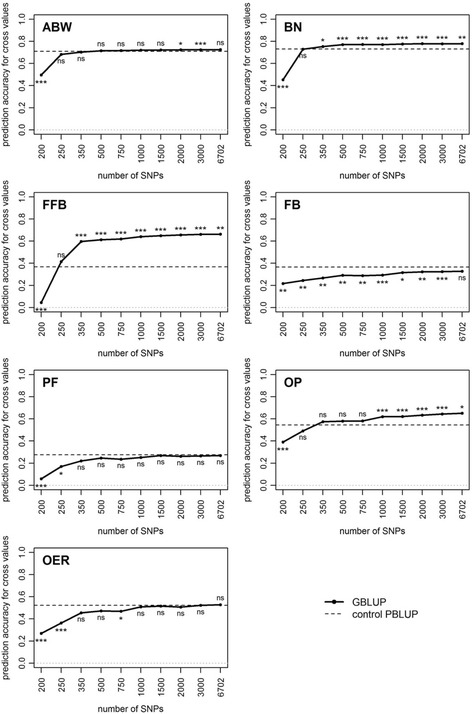
Mean prediction accuracy over six replicates of hybrid crosses, when predicting cross values of Site 2 using a model calibrated on Site 1, using GBLUP (solid line) and the control PBLUP (dashed black line). The last level of the number of SNPs (6702) is the mean number of SNPs in Group A and Group B. Significance of ANOVA for prediction model: *** *P* < 0.001, ** 0.001 ≤ *P* < 0.01, * 0.01 ≤ *P* < 0.05, *ns* not significant. Values are means over *n* = 26 random marker subsets (except when using all the markers, *n* = 1)

Prediction accuracy increased with the number of SNP and reached a plateau starting between 500 SNPs (ABW and BN) and 2000 (FB), depending on the trait. When 200 SNPs were used, the prediction accuracy of the cross values ranged from very low to intermediate (0.04 ± 0.05 for FFB to 0.50 ± 0.12 for ABW) (Fig. 3). For ABW, BN, FFB and OP, with GBLUP, significantly higher prediction accuracies were obtained than prediction accuracies with PBLUP, using at least 2000 SNPs for ABW, 350 for BN and FFB and 1000 for OP (for ABW, the non-significant difference obtained with all SNPs was caused by the reduced power to detect significant differences in this case, resulting from the smaller number of replicates compared with when random subsets of SNPs were made, and from the small magnitude of the difference). The trait for which GBLUP outperformed PBLUP the most was FFB, with GBLUP prediction accuracy of 0.66 when using all SNPs, i.e. 80% higher than with PBLUP, followed by OP (0.65, +19.3%), BN (0.78, +6.7%) and ABW (0.73, +2.3%) (Fig. 3). For PF and OER, GBLUP and PBLUP gave the same prediction accuracies. FB was the only trait where PBLUP was significantly more accurate than GBLUP even when a large number of SNPs was used, as GBLUP prediction accuracy was 10.3% lower than PBLUP.

**Fig. 4:**
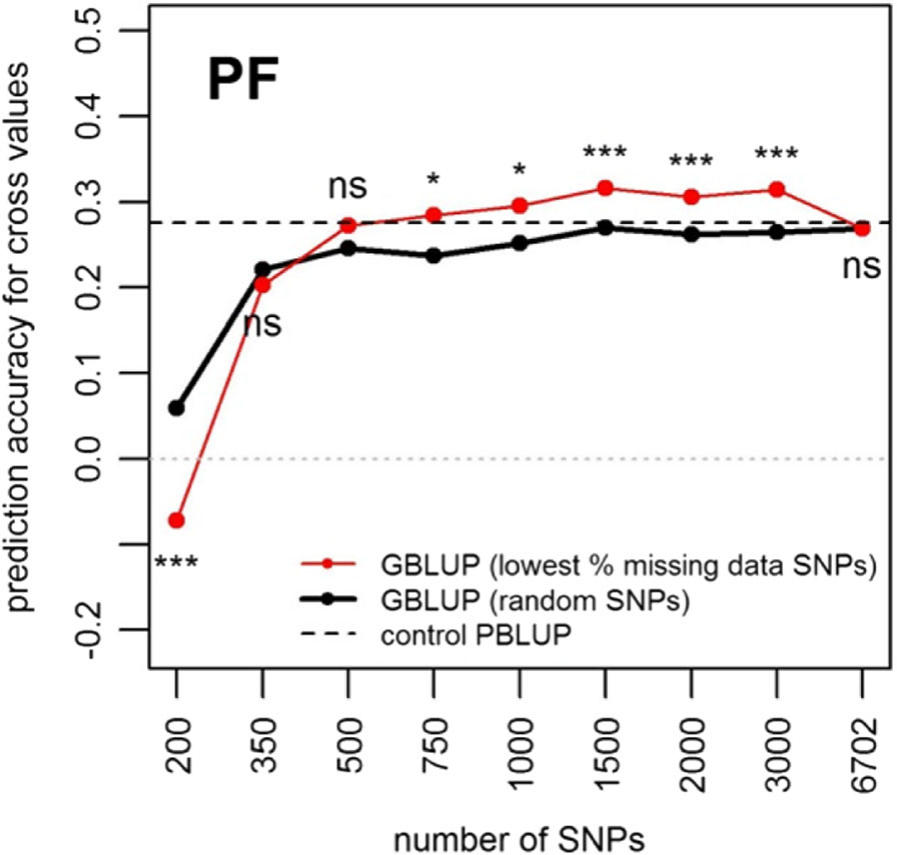
Mean prediction accuracy over six replicates of hybrid crosses, when predicting cross values of Site 2 using a model calibrated on Site 1 for the PF trait, using GBLUP with random subsets of SNPs (solid black line), SNP subsets minimizing the percentage of missing data (red line) and control PBLUP (dashed line). The last level in the number of SNPs (6702) is the mean number of SNPs in Group A and Group B. Significance of ANOVA for method of SNP sampling: *** *P* < 0.001, * 0.01 ≤ *P <* 0.05, *ns* not significant. Values are means over *n* = 26 replicates of random marker subsets, *n* = 3 when minimizing percentage of missing data and *n* = 1 when using all the markers

**Fig. 5:**
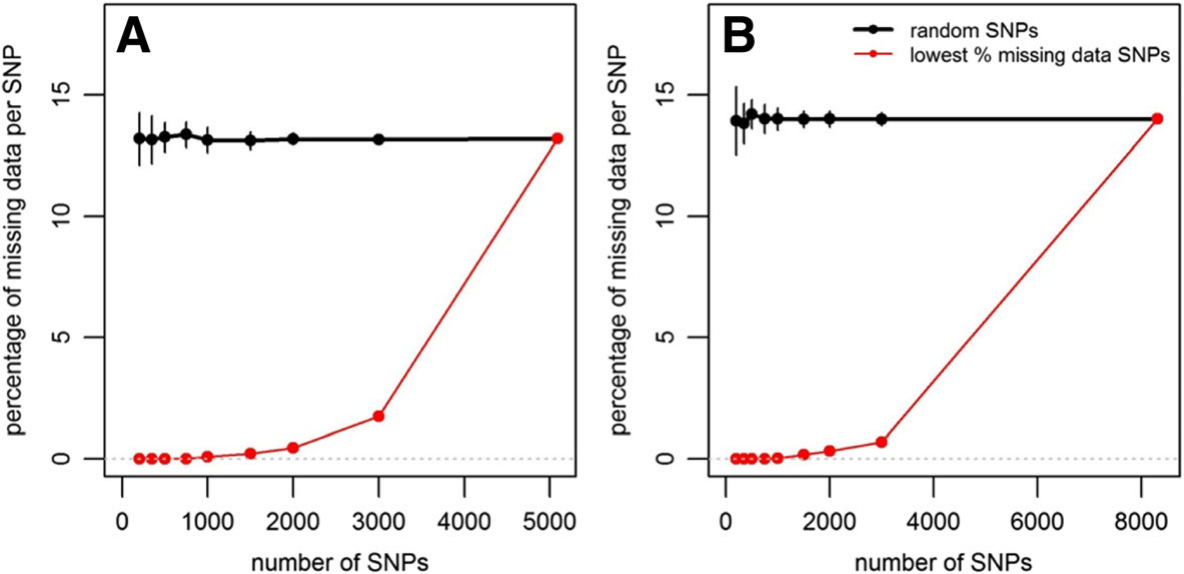
Percentage of missing molecular data in group **a** (left panel) and group **b** (right panel) according to the method used to sample SNPs (random sampling, solid black line, or selecting SNPs with the lowest percentage of missing data, red line). Values are means over *n* = 26 replicates of random marker subsets, *n* = 3 when minimizing percentage of missing data and *n* = 1 when using all the markers. Vertical bars are standard deviations

The variation in GS prediction accuracy for intermediate marker densities indicated that some SNP subsets enabled higher prediction accuracy than when all the markers were used (not shown). Defining subsets of SNPs with the lowest percentage of missing data increased GBLUP prediction accuracy in one trait, PF (Fig. 4). For this trait, using 500 to 3000 SNPs led to GBLUP prediction accuracy higher than when the SNPs were randomly sampled. With 1500 to 3000 SNPs, the increase reached 17.6% and was highly significant (*P* < 0.001). The accuracy of GBLUP with SNP subsets minimizing the percentage of missing data was then also higher than PBLUP accuracy (+13.2%), although the difference was not significant. In the other traits, this method of SNP sampling led to prediction accuracies with 1500 to 3000 SNPs similar to random sampling. The method led to very low percentages of missing data in both parental groups, ranging from 0% to less than 2% when the number of SNPs varied from 200 to 3000 (Fig. 5). The best way to define SNPs for GS was therefore to use at least 2000 SNPs with the lowest percentage of missing data. From Fig. 1, this could be achieved by discarding the SNPs with more than 5% missing data.

**Fig. 6:**
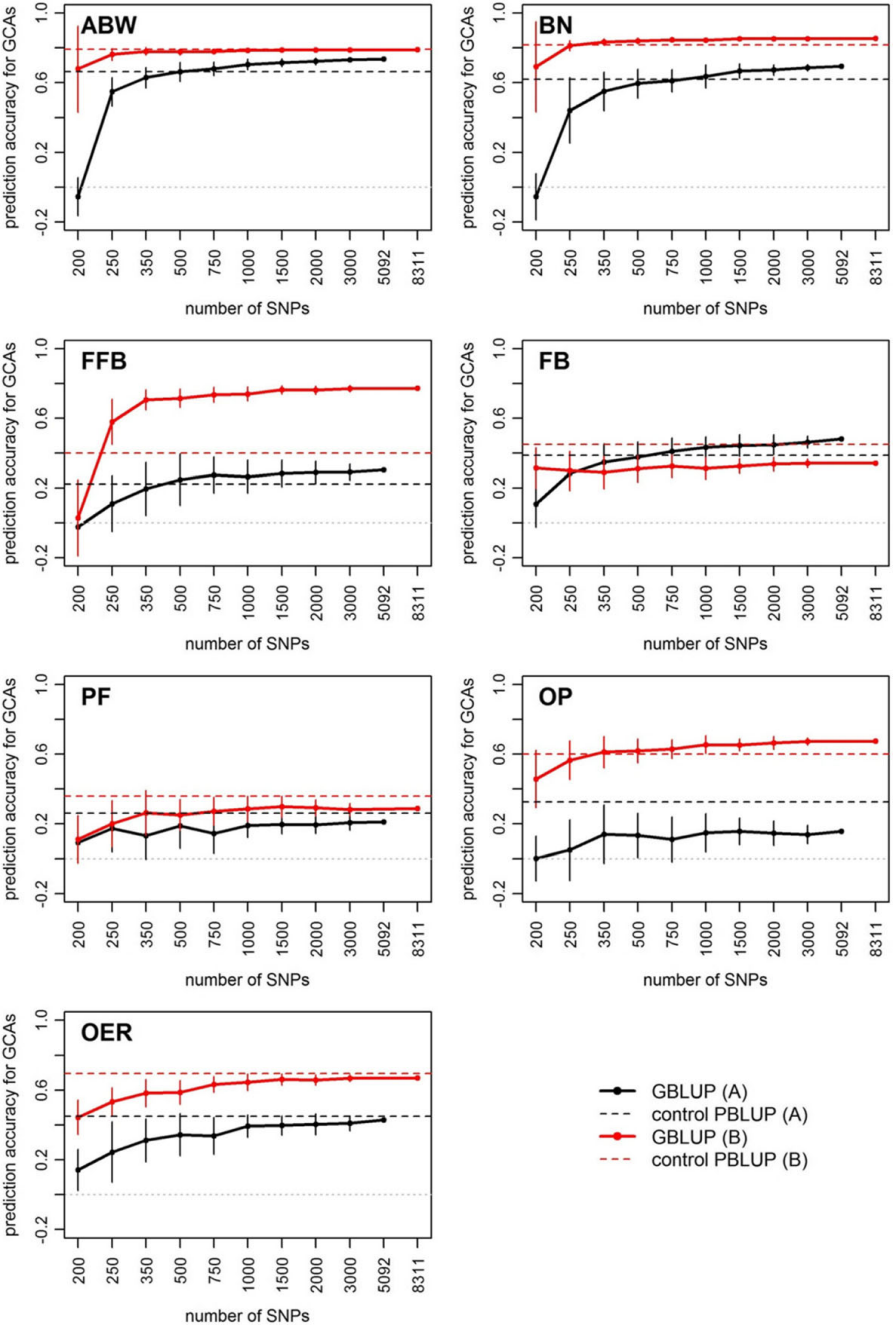
Prediction accuracy when predicting the GCA of parents in Group A (black lines) and Group B (red lines) evaluated in Site 2 using a model calibrated on Site 1, using GBLUP (solid lines) and control PBLUP (dashed lines). Values are means over *n* = 26 random marker subsets (except when using all the markers, *n* = 1). Vertical bars are standard deviations

The GCA prediction accuracies were higher in Group A than in Group B, except for FB (Fig. 6). They ranged from −0.05 to 0.85, depending on the trait, SNP density, and the parental group. They started plateauing at 350 SNPs (OP in Group A, ABW and BN in Group B) to 3000 (ABW, BN and FB in Group A), depending on the trait and parental group. Using all SNPs, they ranged from 0.16 for OP in Group A to 0.85 for BN in Group B. The prediction accuracies of the cross values were usually closer to the GCA prediction accuracies obtained in Group B than in Group A. In particular, the GCA prediction accuracy in Group A for FFB and OP was much lower than in Group B or than the prediction accuracy of the cross values. In Group A, the relative performance of GBLUP and PBLUP in terms of GCAs prediction accuracy was similar to that observed with prediction accuracies of the cross values for most of the traits, except for OP and FB. For OP, GBLUP with all SNPs was much less accurate than PBLUP in predicting GCAs (−52.1%) whereas it was more accurate for cross values. For FB, GBLUP with all SNPs was 24% more accurate than PBLUP for GCAs but less accurate than PBLUP for cross values. In Group B, the relative performance of GBLUP and PBLUP in terms of the prediction accuracy of GCAs was similar to that observed with the prediction accuracies of cross values for all traits.

### Impact of genomic preselection on hybrid performances for FFB

The variances of the true GCAs of FFB of the 67 A and 42 B validation individuals, ${\sigma}_{\mathrm{g}\prime}^2$, were 21.6 in Group A and 56.4 in Group B. For the same individuals, the selection accuracy of the GCAs estimated from the progeny tests, *r*(${\widehat{\mathbf{g}\prime}}_{\mathbf{TBLUP}}$, **g**′), was on average 0.54 in Group A and 0.76 in Group B. The A and B GS prediction accuracies *r*(${\widehat{\mathbf{g}\prime}}_{\mathbf{GBLUP}}$, ${\widehat{\mathbf{g}\prime}}_{\mathbf{TBLUP}}$) obtained from the previously described GS validation with all SNPs were 0.30 for Group A and 0.77 for Group B. μ_FFB_ was 120.9 kg per palm (the other figures used in the variance-covariance matrices are given in Additional file 8: Table S3).

**Fig. 7:**
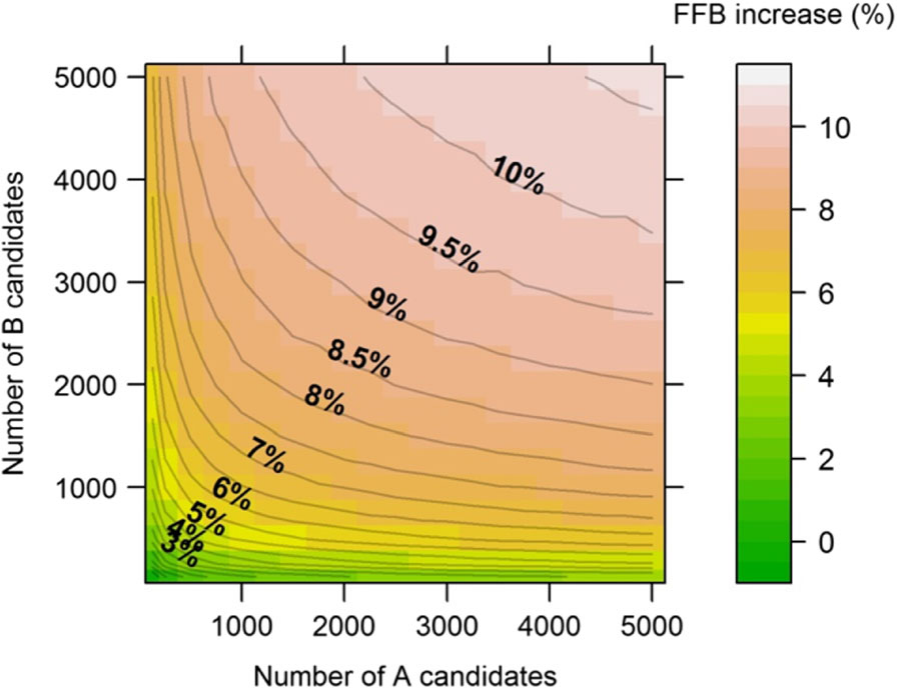
Increase in production of fresh fruit bunches (FFB) in the crosses selected in Site 2 progeny tests if preliminary genomic preselection had been applied in the parental populations. The increase in FFB is expressed as a percentage of the FFB performance of the hybrids selected using the current method (with no genomic preselection). Axes show the number of A and B selection candidates subjected to genomic preselection. Values are means over 20,000 replicates

The simulation based on these empirical results showed that the FFB value of the selected hybrids could have been over 10% higher if A and B candidates had been subjected to genomic preselection for this trait prior to progeny tests, compared to the actual method used, i.e. progeny testing random individuals (Fig. 7). Indeed, the mean FFB of the selected hybrids with conventional RRS was on average 135.9 kg, while with genomic preselection it reached 150.3 kg (+10.6%), when genomic preselection was applied to 5000 A candidates and 5000 B candidates. As expected, the magnitude of the increase was affected by the number of A and B individuals subjected to preselection, although the number of B candidates had a greater effect than the number of A candidates. Thus, with a fixed number of 1000 A candidates subjected to genomic preselection, increasing the number of B candidates from 125 to 5000 increased the FFB from 2.4% to 9.4%, and this would have been even higher if a larger B population had been used. By contrast, with 1000 B candidates for genomic preselection, increasing the number of A candidates from 125 to 5000 only increased the FFB from 4% to 7.7%.

## Discussion

### Toward the use of genomic selection

We found that a reciprocal recurrent genomic selection (GS) breeding scheme can be implemented in oil palm, applying a genomic preselection among parental populations to identify individuals with highest genetic value in hybrid crosses, before progeny testing them to make the final selection on all traits. Genomic preselection increases the genetic gain compared to the current RRS breeding scheme thanks to higher selection intensity. GS can be used for several key components of palm oil yield, in particular bunch production and the percentage of oil in the mesocarp. This required at least 2000 SNPs, with best results achieved when only SNPs with less than 5% missing data were used, imputed taking into account pedigree information. We illustrated the impact of genomic preselection on the bunch production trait (FFB) and showed that a preliminary genomic preselection could have further increased (by >10%) the FFB performance of the hybrids selected from Site 2, compared to the actual method with no preselection for this trait.

The efficiency of the genomic preselection observed for several traits resulted from two facts. First, the GS prediction accuracies obtained here when predicting the genetic values of unobserved hybrid crosses and their parents could reach intermediate to high levels (> 0.6). Second, the GS prediction accuracies could be significantly higher than the prediction accuracies of the pedigree-based control model (PBLUP), indicating that GS was able to capture genetic differences within full-sib families of parents (i.e. the Mendelian segregation terms) in addition to genetic differences between parental families. In this case, GS enables identification of the best individuals of the best families, as currently done among the progeny tested individuals.

According to the results of the simulation, the extra gain in FFB of selected hybrids obtained by using genomic preselection reached, when for instance 1000 A candidates and 2000 B candidates were genotyped, 10.5 kg palm^−1^ year^−1^ over a 3–7 year period. With a planting density of 143 individuals ha^−1^ and applying a correction of 95% standing palms, this extra gain would be 1.43 t ha^−1^ year^−1^. Assuming a mill OER (oil extraction rate) value of the selected hybrids of 28%, based on the current performance of similar material sold by PalmElit, this represents and extra oil production of 400 kg ha^−1^. Finally, considering a crude palm oil price of 640 US$ t^−1^ (mean price in 2016, www.indexmundi.com), we were able to estimate the economic gain of genomic preselection for growers: in the conditions of Site 2 experiment, genomic preselection on FFB with the genotyping of 3000 individuals would have increased incomes by 256 US$ ha^−1^ year^−1^ on average over the 3–7 year production period in plantations with hybrids selected from this experiment, compared to the conventional breeding approach that was actually applied. Considering that most oil palm breeding companies are also large scale palm oil producers with tens of thousands of hectares or more, and given the GBS price per sample, the associated extra genotyping costs would be quickly recovered by the increase in annual income per hectare. The question of the additional gain that would be obtained with conventional RRS by using the extra cost of GS to progeny test more A and B parents remains, but given the much higher costs of progeny tests compared to the GBS price, the increase in selection intensity would likely be negligible compared to what can be achieved with GS.

This study allowed us to extend the promising empirical results of Cros et al. [17] and Marchal et al. [18] in oil palm by using independent validation, larger training sets, a high throughput genotyping approach and more complex validation populations (in particular including progeny of the training parents), and by predicting cross values in addition to parental GCAs. The prediction accuracies obtained here are therefore more relevant for the practical implementation of the method than the previous prediction accuracies, and as a result, it was possible to use them to obtain a realistic first empirical estimate of the additional genetic gain that could be obtained with GS in oil palm.

### Research required to reach the full potential of GS

The additional gain obtained here thanks to genomic preselection compared to the conventional method is close to the values obtained in hybrid cereals by Marulanda et al. [7] when they compared the standard phenotypic scheme with an alternative scheme with genomic preselection. In their case, achieving more benefits from GS required adopting a genomic breeding scheme with fewer stages of phenotypic selection in order to reduce the duration of the breeding cycle. Developing an efficient breeding scheme with a reduced generation interval thanks to GS is also desirable in oil palm. Indeed, the genomic preselection suggested here is not the optimal use of the possibilities offered by GS for this species, i.e. reducing the generation interval by not implementing progeny tests in some generations [15, 16], as well as increasing selection intensity for all traits. We conclude that for now, the use of GS should be limited to preselection for some traits before progeny tests because GS did not perform sufficiently well for all the traits evaluated during the progeny tests. Indeed, the fact that a control model using pedigrees instead of marker data (PBLUP) gave prediction accuracies equal or even higher than GS for some traits indicated that, for these traits, GS was not able to take the Mendelian segregation into account. Hence, it does not enable selection within full-sib families of parents, while this is the core of oil palm breeding. Other studies are therefore required to increase GS prediction accuracies for all yield components, which would enable its optimal use. In particular, this could be done by increasing the size of the training set. One efficient way to reach this goal would be to use a training set aggregating data from multiple breeding cycles, as demonstrated empirically in hybrid rye by Auinger et al. [52]. Also, because the parents of the oil palm hybrid crosses are heterozygotes, another tempting approach would be to genotype hybrid individuals in addition to their parents, as suggested by Cros et al. [16] and recently evaluated by Kwong et al. [19]. In addition to increasing the size of the training set, optimizing its design is a further way to achieve higher GS accuracy. Several approaches have been developed [53, 54, 55], in particular for hybrid crops [56], which should now be investigated in detail in oil palm.

We noted that PBLUP prediction accuracy could be higher for some traits (ABW and BN), as previously observed in Cros et al. [17], leaving little room for improvement by GBLUP. This indicated that, for these traits, the available dataset was not optimal for GS validation. Indeed, it is easier to show the GBLUP ability to account for the Mendelian segregation term when the phenotypic variability is not structured by differences between families. With such a structure, the ability of GBLUP to account for the Mendelian segregation term would be better evaluated by measuring prediction accuracy in large full-sib families of A and B parents. To this end, suitable experiments should be conducted by progeny testing a significant number of A or B individuals of several full-sib families.

Here we considered rather similar environments, while in real situations the marketing area of the best crosses could possibly involve more contrasted environments. In that case, particularly if genotype by environment interactions exist, it would be necessary to use models combining phenotypic data from various environments, genomic data and environmental covariables (see for instance Bustos-Korts et al. [57]). However, this is an area that requires further methodological investigation, as well as access to multi-environment oil palm data that are currently not available.

### Genomic information

The SNP density obtained with GBS was sufficiently high to achieve maximum prediction accuracy with our dataset. However, with a larger or more diverse training set, it might be necessary to use a larger number of SNPs than the number that corresponded to plateaus in our study. This would likely not be a problem with GBS, as it would yield more markers if applied to a larger or more diverse population. This confirms the usefulness of GBS for GS already noted in other species [23]. In oil palm, GBS appears to be an efficient genotyping approach for genetics studies in general, as it has already been successfully used for mapping and QTL detection [25].

Marchal et al. [18] studied the effect of SSR density on GCA accuracy for ABW and BN traits. In their study (like in ours) accuracy plateaued at similar marker density for the two traits, and Group A required more markers (160 SSRs or 3000 SNPs) than Group B (90 SSRs or 350 SNPs). The higher number of SNPs compared to SSRs had two origins. First, SNP markers were less informative due to their biallelic nature, than SSRs. Indeed, the SSRs used by Marchal et al. [18] had on average 3.1 alleles per SSR in Group A and 6.3 in Group B, and were therefore more polymorphic than SNPs. Second, Marchal et al. [18] predicted the GCA of progeny tested individuals, while we only considered individuals with no phenotypic data record. As a consequence, in Marchal et al. [18], the prediction of the GCAs used the genomic coancestries between the individuals and the phenotypic data of their hybrid progenies, while in our study, the phenotypic data were those of the progenies of the training individuals, which were comparatively less informative and therefore required more markers.

We found that using the pedigrees when imputing the missing SNP data enabled an increase in prediction accuracy. This indicated that without pedigree information, BEAGLE 4.0 imputed genotypes could actually be inconsistent with the parental genotypes, and that the weight of those erroneous genotypes was high enough to significantly decrease GS accuracy in some traits.

The observed variation in GBLUP predictive ability between SNP samples led us to apply several filtering strategies, in addition to the method minimizing the percentage of missing data presented here: most even genome coverage, lowest linkage disequilibrium (LD) between SNPs, smallest departure from the Hardy-Weinberg equilibrium [58] and highest MAF [59]. We do not present the results here because these methods had inconsistent or detrimental effects on prediction accuracy. The fact that filtering SNPs according to the percentage of missing data was able to increase accuracy was also reported by Jarquín et al. [59]. However, these authors also found that the accuracy of GS could be increased by filtering SNPs based on MAF, while this was not the case in our study. One possible explanation is that our population was more complex, with a more unbalanced contribution of the population founders, which could have led to the existence of low frequency alleles but that were representative of some families or individuals, and therefore that were useful to keep in the dataset. This might also result from the highest mean depth per SNP in our study (around 70 versus 11 in the study by Jarquín et al.), making the minor alleles identified here less likely to be sequencing errors, and therefore reducing the usefulness of discarding the SNPs with low MAF. The fact that filtering SNPs based on the percentage of missing data was only efficient for PF suggested a problem with the imputation of markers located in genome regions of importance for this trait and that had a high percentage of missing data. An alternative solution to these imputation and filtering problems would be to use a SNP array instead of GBS, as the percentage of missing data in SNP arrays is very low. This could be done with already available arrays [19, 60, 61], or by developing a new array more specific to the populations used here.

In addition to these SNP filtering strategies, we also used several methods to compute the genomic coancestry matrices **G**, to check whether they could improve GS accuracies: applying corrections to **G** to account for the specificities of GBS (heterogeneous sequencing depth, relatively high percentage of missing data) [58, 62], and computing **G** matrices from SNPs weighted according to local LD levels [63, 64]. These results are not presented here as, with our data, they did not improve prediction accuracies.

According to their pedigrees, the individuals used for validation were more related to the training individuals in Group B than in Group A, but the SNPs showed they were actually related to the same degree. This likely resulted from the fact that the pedigree of Group A did not go back to the founders, whereas it did (or almost did) for Group B, and because SNPs are able to capture these relationships even when they do not appear in the pedigrees due to their incompleteness. This was already observed in our previous study [17] using SSR markers.

### Variations among traits and parental groups

Our results show that the phenotypic distribution of the traits affected GS accuracy. Muranty et al. [65] also observed in apple that accuracy was strongly affected by phenotypic distribution, with traits for which GS performed poorly often having reduced phenotypic variation and skewed distribution. Both studies therefore are partly in agreement, because in our study, GS accuracy only appeared to be related to phenotypic variation and not to the skewness of the distribution. Indeed, the two traits with the lowest GS cross value prediction accuracies, FB and PF, also had low phenotypic variation. Regarding skewness, FB had the least skewed distribution, and ABW had the most skewed distribution but high GS accuracy for cross value. However, OP had a similarly low phenotypic variation as FB and PF, but had a prediction accuracy of cross values >0.6. Therefore, other factors, such as the genetic architecture of the traits (number of genes, distribution of their effects, etc.), must affect accuracy, even if they do not affect phenotypic distribution.

The smaller variance of true GCAs in Group A compared to Group B was expected from their respective history. As a consequence, the main driver of the prediction accuracy of cross values was the prediction accuracy of GCA in Group B. Likewise, increasing the number of B candidates was a more efficient way to increase the FFB of hybrid crosses than increasing the number of A candidates.

### Prediction of dominance effects

The fact that adding an SCA term to the parental GCAs when calculating the prediction accuracies of cross values did not improve the results was likely the consequence of an insufficient proportion of dominance variance in total genetic variance. Indeed, some traits e.g. ABW, BN and FFB, had particularly low SCA variance, for which it was almost null. This low dominance variance might seem inconsistent with the use of RRS in oil palm breeding justified by heterosis in FFB [10], but this rather indicates that this heterosis is a general phenomenon occurring in the case of A × B crosses, with a magnitude that varies only slightly between crosses. For the other traits, even if the proportion of SCA variance in the total variance could be much higher, it might still not be high enough to increase prediction accuracy when SCA terms are predicted in addition to GCAs. Indeed, Denis and Bouvet [66] showed in simulations that including dominance effects in the GS model was only advantageous when dominance effects were preponderant (dominance to additive variance ratio of 1). Similar results were obtained empirically in apple [67] and rice [68], where dominance effects did not improve prediction accuracies.

## Conclusion

GS prediction accuracies reached intermediate to high values for some key yield components (0.60–0.85), in particular for the production of fresh fruit bunches (FFB) and the oil-to-pulp ratio, using at least 2000 SNPs with less than 5% missing data, imputed using pedigrees. This enabled genomic preselection in the parental populations prior to progeny tests, which increased selection intensity. We show this for FFB, for which parental genomic preselection could have increased the FFB performance of the selected hybrids by more than 10%, compared to the current method without preselection for this trait. Preselection for key yield components using GBS is the first possible application of GS in oil palm for which empirical evidence of efficiency is available.

However, more benefits are expected from GS in this species. Indeed, the genomic preselection suggested here would not make it possible to increase selection intensity for all traits, and progeny tests would still be required, making it impossible to reduce the generation interval. Further research is therefore needed to enhance GS prediction accuracies for all yield components. In particular, further studies are necessary to enlarge and optimize the training set and to model genotype by environment interactions. This would enable the optimal use of GS, which would revolutionize oil palm breeding. GS clearly has a major role to play in meeting the huge increase in the demand for palm oil expected in the coming decades, in a sustainable way, i.e. by maximizing the productivity of the existing planted area.

## Additional files

Additional file 1: Table S1. Characteristics of the experimental designs used in the two sites. Distribution of progeny tested individuals among parental groups and breeding populations. (DOCX 19 kb)

Additional file 2: Figure S1. Pedigree of Group A individuals progeny tested in Site 1 (training set, blue) and progeny tested in Site 2 and genotyped (validation set, red *n* = 67) or common to both sites (green). (DOCX 51 kb)

Additional file 3: Figure S2. Pedigree of Group B progeny tested individuals in Site 1 (training set, blue), progeny tested individuals in Site 2 and genotyped (validation set, red *n* = 43) or common to both sites (green). (DOCX 50 kb)

Additional file 4: Table S2. Tassel v5.2.29 GBS pipeline used to process raw sequence data. (DOCX 13 kb)

Additional file 5: Figure S3. Physical map of the 5092 SNPs available for Group A. The figures below the chromosomes indicate their number of SNPs. The colors indicate the number of SNPs per segments of chromosomes of 1,000,000 bp. (DOCX 153 kb)

Additional file 6: Figure S4. Physical map of the 8311 SNPs available for Group B. The figures below the chromosomes indicate their number of SNPs. The colors indicate the number of SNPs per segments of chromosomes of 1,000,000 bp. (DOCX 156 kb)

Additional file 7: Figure S5. Method overview for estimation of prediction accuracies. (DOCX 26 kb)

Additional file 8: Table S3.Variance-covariance matrices between the true (**g** ^′^), reference (${\widehat{\mathbf{g}\prime}}_{\mathbf{TBLUP}}$) and genomic estimated GCAs (${\widehat{\mathbf{g}\prime}}_{\mathbf{GBLUP}}$) used to simulate the 5000 individuals comprising the populations of selection candidates. (DOCX 12 kb)

## Abbreviations

ABW: average bunch weight
BN: annual cumulative bunch number
FB: fruit-to-bunch ratio
FFB: fresh fruit bunch
GBLUP: genomic best linear unbiased predictor
GBS: genotyping-by-sequencing
GCA: general combining ability
GS: genomic selection
OER: oil extraction rate
OP: oil-to-pulp ratio
PBLUP: pedigree-based best linear unbiased predictor
PF: pulp-to-fruit ratio
RRS: reciprocal recurrent selection
SCA: specific combining ability
SNP: single nucleotide polymorphism
TBLUP: traditional best linear unbiased predictor
