# Supplementary material for: Genomic preselection with genotyping-by-sequencing increases performance of commercial oil palm hybrid crosses
Source: BMC Genomics. 2017 Nov 2;18:839. doi: 10.1186/s12864-017-4179-3 (PMC5667528; doi:10.1186/s12864-017-4179-3)
Supplement: Supplementary file 1 — Characteristics of the experimental designs used in the two sites. Distribution of progeny tested individuals among parental groups and breeding populations. (DOCX 19 kb) [file 12864_2017_4179_MOESM1_ESM.docx]

Additional file 1: Table S1 Distribution of progeny tested individuals among parental groups and breeding populations

|  | Status | | | | | |
| --- | --- | --- | --- | --- | --- | --- |
|  | **Site 1** | Site 1 | Site 1 | Site 2 | Site 2 | **Site 2** |
|  |  | only | only, |  | only | **only,** |
|  |  |  | genotyped |  |  | **genotyped** |
| **Parental Group A** |  |  |  |  |  |  |
| Angola | **11** | 11 | 7 | 0 | 0 | **0** |
| Angola × Deli | **0** | 0 | 0 | 17 | 17 | **7** |
| Deli | **139** | 126 | 111 | 123 | 110 | **60** |
| *Total* | ***150*** | *137* | *118* | *140* | *127* | ***67*** |
|  |  |  |  |  |  |  |
| **Parental Group B** |  |  |  |  |  |  |
| Cameroon | **0** | 0 | 0 | 3 | 3 | **0** |
| Cameroon × AVROS | **0** | 0 | 0 | 15 | 15 | **0** |
| Lisombe Kinshasa | **8** | 7 | 1 | 4 | 3 | **1** |
| Lisombe Kinshasa × La Mé | **0** | 0 | 0 | 3 | 3 | **0** |
| La Mé | **112** | 105 | 88 | 37 | 30 | **18** |
| La Mé × Yangambi / Sibiti | **7** | 6 | 6 | 11 | 10 | **4** |
| Nigeria | **0** | 2 | 0 | 3 | 0 | **0** |
| Nigeria × La Mé | **0** | 0 | 0 | 7 | 7 | **0** |
| Nigeria × Yangambi | **5** | 0 | 2 | 3 | 3 | **4** |
| Yangambi | **24** | 22 | 18 | 21 | 19 | **15** |
| *Total* | ***156*** | *142* | *115* | *107* | *93* | ***42*** |
|  |  |  |  |  |  |  |
| **Site 1, bold**: parents of training crosses, **Site 2, bold**: used to compute prediction accuracy when predicting GCAs. ‘Status’ shows the location of the hybrid crosses involving the corresponding parents (Site 1, Site 2 or both; genotyped or not) | | | | | | |

Table S1 Characteristics of the experimental designs used in the two sites

|  |  | Site 1 | | Site 2 | |
| --- | --- | --- | --- | --- | --- |
|  |  | Production | Bunch quality | Production | Bunch quality |
| **Hybrid crosses** |  |  |  |  |  |
| Number |  | **497 ^(1)^** | **487 ^(1)^** | 433 | 393 |
| Number not tested in the other site |  | 488 | 478 | 424 | 384 |
| Number not tested in the other site with genotyped parents |  |  |  | **199 ^(2)^** | **198 ^(2)^** |
| Mean number per progeny tested individuals (min-max) | A | 3.1 (1-8) | 3.3 (1-8) | 3.1 (1-18) | 3.2 (1-16) |
|  | B | 3.2 (1-8) | 3.1 (1-8) | 4.0 (1-26) | 3.8 (1-25) |
| **Progeny tested individuals** |  |  |  |  |  |
| Number | A | **150 ^(1)^** | **149 ^(1)^** | 140 | 123 |
|  | B | **156 ^(1)^** | **156 ^(1)^** | 107 | 103 |
| Number genotyped | A | 130 | 129 | 79 | 77 |
|  | B | 127 | 127 | 54 | 54 |
| Number genotyped and not tested in the other site | A | 118 | 119 | **67 ^(3)^** | **67 ^(3)^** |
|  | B | 115 | 115 | **42 ^(3)^** | **42 ^(3)^** |
| **Hybrid individuals** |  |  |  |  |  |
| Number |  | 32,656 | 21,917 | 27,229 | 19,180 |
| Mean number per cross (min-max) |  | 66 (22-507) | 45 (16-292) | 63 (20-779) | 49 (17-493) |
| Mean number per progeny tested individual (min-max) | A | 218 (26-758) | 147 (25-491) | 195 (23-1,811) | 156 (20-1,282) |
|  | B | 209 (24-891) | 141 (24-508) | 255 (24-2,592) | 186 (19-1,772) |
| Mean number of bunches analyzed per evaluated hybrid individual |  |  | 2.6 (1-8) |  | 1.7 (1-11) |
| **Trials** |  |  |  |  |  |
| Number |  | 30 | 27 | 22 | 20 |
| Planting years |  | 1986 and 1995 to 2003 | | 2005 to 2010 | |
|  |  |  | |  | |

**^(1)^** used to train the genomic model (training set)

**^(2)^** used to compute prediction accuracy when predicting cross values (validation set)

**^(3)^** used to compute prediction accuracy when predicting parental GCAs (validation set)
