# Supplementary material for: Genomic preselection with genotyping-by-sequencing increases performance of commercial oil palm hybrid crosses
Source: BMC Genomics. 2017 Nov 2;18:839. doi: 10.1186/s12864-017-4179-3 (PMC5667528; doi:10.1186/s12864-017-4179-3)
Supplement: Supplementary file 3 — Pedigree of Group B progeny tested individuals in Site 1 (training set, blue), progeny tested individuals in Site 2 and genotyped (validation set, red n = 43) or common to both sites (green). (DOCX 50 kb) [file 12864_2017_4179_MOESM3_ESM.docx]

Additional file 3: Figure S2
