# Supplementary material for: Genomic preselection with genotyping-by-sequencing increases performance of commercial oil palm hybrid crosses
Source: BMC Genomics. 2017 Nov 2;18:839. doi: 10.1186/s12864-017-4179-3 (PMC5667528; doi:10.1186/s12864-017-4179-3)
Supplement: Supplementary file 4 — Tassel v5.2.29 GBS pipeline used to process raw sequence data. (DOCX 13 kb) [file 12864_2017_4179_MOESM4_ESM.docx]

Additional file 4: Table S2 Tassel v5.2.29 GBS pipeline used to process raw sequence data

| Step_plugin | Parameters | Results | Value | % |
| --- | --- | --- | --- | --- |
| 00 (raw fastq data) |  | Number of reads in lanes | 238,493,056 |  |
| 01_GBSSeqToTagDB | ePstI c20 kmerL68 minKmerL20 mnQS20 | Number of correct barcoded reads | 152,020,019 | 63.7 |
| 01_GBSSeqToTagDB | ePstI c20 kmerL68 minKmerL20 mnQS20 | Number of tags | 476,589 |  |
| 02_TagExportToFastq | c1 | Export tags to fastq | 476,589 |  |
| 03_BowtieToSAM | very-sensitive-local | Number of tags aligned once | 243,794 | 51.2 |
| 03_BowtieToSAM | very-sensitive-local | Number of tags aligned >1 time | 77,160 | 16.2 |
| 04_SAMToGBSdb | aProp0 aLen0 | Number of mapped tags | 320,954 | 67.3 |
| 05_DiscoverySNPCaller | maxTagsCutSite68 mnLCov0.1 mnMAF0.0025 eR 0.01 | Number of polymorphic sites | 109,201 |  |
| 05_DiscoverySNPCaller | maxTagsCutSite68 mnLCov0.1 mnMAF0.0025 eR 0.01 | Number of alleles | 230,100 |  |
| 06_SNPQualityProfiler |  | Number of polymorphic sites | 109,201 |  |
| 07_ProdSNPCaller | ePstI kmerL68 mnQS0 | Number of polymorphic sites | 109,201 |  |
