# Supplementary figures and images for: Genomic preselection with genotyping-by-sequencing increases performance of commercial oil palm hybrid crosses
Source: BMC Genomics. 2017 Nov 2;18:839. doi: 10.1186/s12864-017-4179-3 (PMC5667528; doi:10.1186/s12864-017-4179-3)

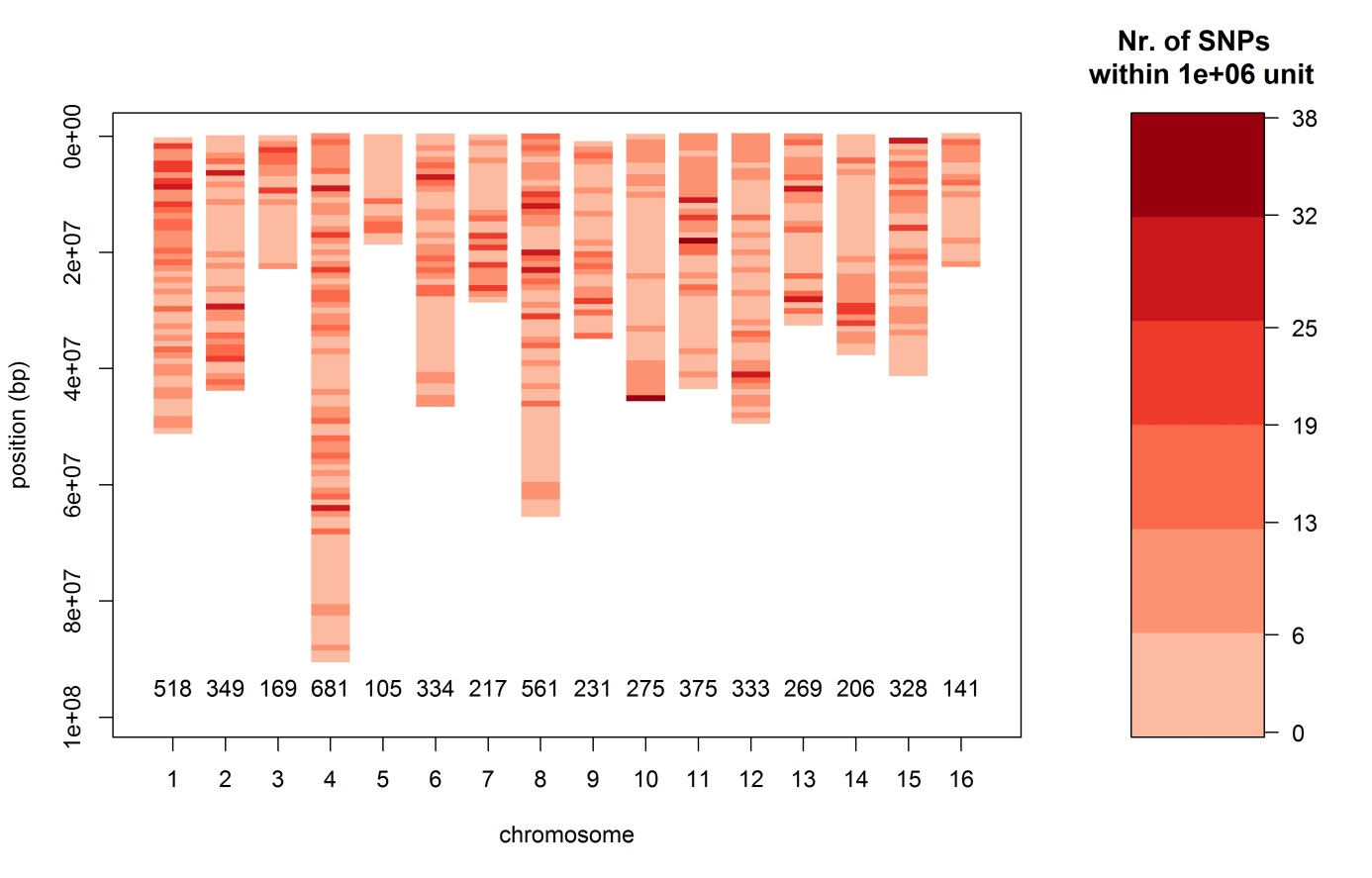


Additional file 5: Figure S3

Supplement: Supplementary file 5 — Physical map of the 5092 SNPs available for Group A. The figures below the chromosomes indicate their number of SNPs. The colors indicate the number of SNPs per segments of chromosomes of 1,000,000 bp. (DOCX 153 kb) [file 12864_2017_4179_MOESM5_ESM.docx]

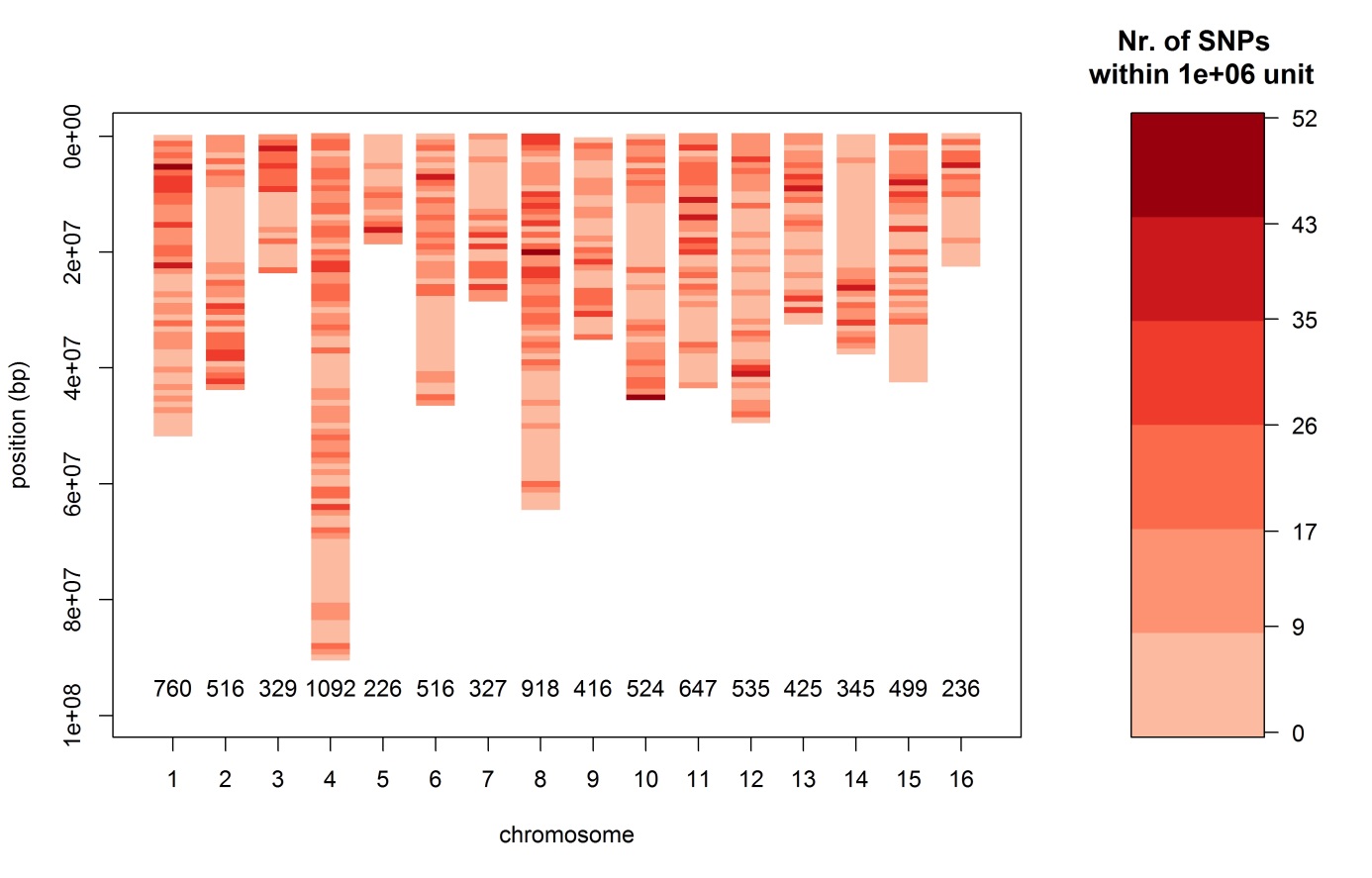


Additional file 6: Figure S4

Supplement: Supplementary file 6 — Physical map of the 8311 SNPs available for Group B. The figures below the chromosomes indicate their number of SNPs. The colors indicate the number of SNPs per segments of chromosomes of 1,000,000 bp. (DOCX 156 kb) [file 12864_2017_4179_MOESM6_ESM.docx]
