# Supplementary material for: Genomic preselection with genotyping-by-sequencing increases performance of commercial oil palm hybrid crosses
Source: BMC Genomics. 2017 Nov 2;18:839. doi: 10.1186/s12864-017-4179-3 (PMC5667528; doi:10.1186/s12864-017-4179-3)
Supplement: Supplementary file 7 — Method overview for estimation of prediction accuracies. (DOCX 26 kb) [file 12864_2017_4179_MOESM7_ESM.docx]

497 A × B crosses

***SITE 1***

***SITE 2***

***Predictive model:***

- GBLUP

- control PBLUP

***Molecular data:***

- +5,000 SNPs

***Parameters:***

- missing SNP imputation with / without pedigree

- SNP filtering on % missing data

- SNP number

- predictive model with / without SCA

***Prediction of cross values and parental GCAs***

199 A × B crosses

(6 replicates)

**Additional file 7:** **Figure S5**
