# Supplementary material for: Genomic preselection with genotyping-by-sequencing increases performance of commercial oil palm hybrid crosses
Source: BMC Genomics. 2017 Nov 2;18:839. doi: 10.1186/s12864-017-4179-3 (PMC5667528; doi:10.1186/s12864-017-4179-3)
Supplement: Supplementary file 8 — Variance-covariance matrices between the true (g ′), reference (${\widehat{\mathbf{g}\prime}}_{\mathbf{TBLUP}}$g′^TBLUP) and genomic estimated GCAs (${\widehat{\mathbf{g}\prime}}_{\mathbf{GBLUP}}$g′^GBLUP) used to simulate the 5000 individuals comprising the populations of selection candidates. (DOCX 12 kb) [file 12864_2017_4179_MOESM8_ESM.docx]

Additional file 8: Table S3 Variance-covariance matrices between the true ($\mathbf{g'}$), reference (${\hat{\mathbf{g'}}}_{\mathbf{TBLUP}}$) and genomic estimated GCAs (${\hat{\mathbf{g'}}}_{\mathbf{GBLUP}}$) used to simulate the 5,000 individuals comprising the populations of selection candidates

| Group A | Group B |
| --- | --- |
| $\mathbf{g}'$ ${\hat{\mathbf{g'}}}_{\mathbf{TBLUP}}$ ${\hat{\mathbf{g'}}}_{\mathbf{GBLUP}}$  $\mathbf{g}'$ 21.6 6.3 4.8  ${\hat{\mathbf{g'}}}_{\mathbf{TBLUP}}$ 6.3 6.3 1.4  ${\hat{\mathbf{g'}}}_{\mathbf{GBLUP}}$ 4.8 1.4 3.4 | $\mathbf{g}'$ ${\hat{\mathbf{g'}}}_{\mathbf{TBLUP}}$ ${\hat{\mathbf{g'}}}_{\mathbf{GBLUP}}$  $\mathbf{g}'$ 56.4 32.6 39.6  ${\hat{\mathbf{g'}}}_{\mathbf{TBLUP}}$ 32.6 32.6 22.9  ${\hat{\mathbf{g'}}}_{\mathbf{GBLUP}}$ 39.6 22.9 27.1 |
